# Poly[aqua­bis(μ_4_-naphthalene-1,4-di­carboxyl­ato)(1,10-phenanthroline-5,6-dione)dimanganese(II)]

**DOI:** 10.1107/S1600536809046388

**Published:** 2009-11-07

**Authors:** Fang-Di Cong, Feng-Yang Yu, Zhen Wei, Seik Weng Ng

**Affiliations:** a Department of Basic Science, Tianjin Agricultural University, Tjianjin 300384, People’s Republic of China; b Department of Chemistry, University of Malaya, 50603 Kuala Lumpur, Malaysia

## Abstract

The three-dimensional coordination polymer, [Mn_2_(C_12_H_6_O_4_)_2_(C_12_H_6_N_2_O_2_)(H_2_O)]_*n*_, features a water-coord­inated Mn^II^ ion and an N-heterocycle-chelated Mn^II^ ion, both in six-coordinate octa­hedral geometries. Of the two rigid dianions, one is bonded to four Mn^II^ ions, with each of the O atoms being connected to a different metal ion. The other dianion uses one carboxyl­ate group to chelate to one Mn^II^ ion and its other carboxyl­ate group to bind to two Mn^II^ ions.

## Related literature

For similiar manganese naphthalene-1,4-dicarboxyl­ate polymers, see: Boeckmann *et al.* (2009 ▶); Li *et al.* (2008 ▶).
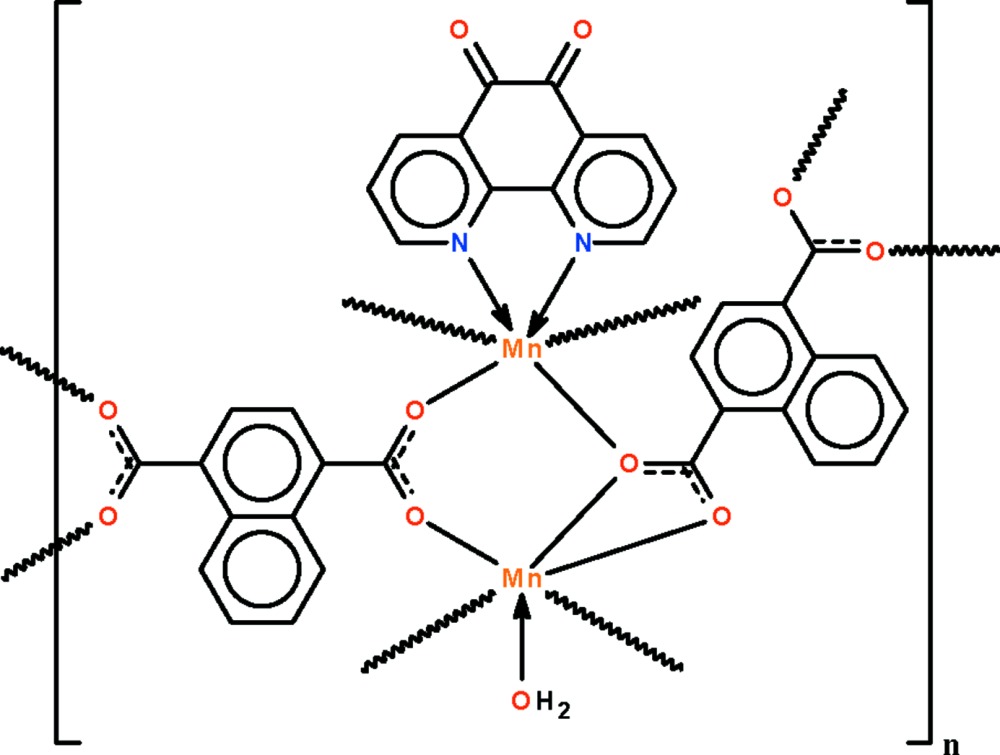


## Experimental

### 

#### Crystal data

[Mn_2_(C_12_H_6_O_4_)_2_(C_12_H_6_N_2_O_2_)(H_2_O)]
*M* *_r_* = 766.42
Monoclinic, 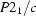

*a* = 8.4393 (8) Å
*b* = 19.2477 (18) Å
*c* = 19.2504 (19) Å
β = 101.781 (1)°
*V* = 3061.1 (5) Å^3^
*Z* = 4
Mo *K*α radiation
μ = 0.90 mm^−1^
*T* = 293 K
0.27 × 0.26 × 0.20 mm

#### Data collection

Bruker APEXII area-detector diffractometer
Absorption correction: multi-scan (*SADABS*; Sheldrick, 1996 ▶) *T*_min_ = 0.794, *T*_max_ = 0.841
16986 measured reflections
6015 independent reflections
4853 reflections with *I* > 2σ(*I*)
*R*_int_ = 0.025

#### Refinement

*R*[*F* ^2^ > 2σ(*F* ^2^)] = 0.036
*wR*(*F* ^2^) = 0.096
*S* = 1.03
6015 reflections
466 parameters
3 restraints
H atoms treated by a mixture of independent and constrained refinement
Δρ_max_ = 0.40 e Å^−3^
Δρ_min_ = −0.24 e Å^−3^

### 

Data collection: *APEX2* (Bruker, 2007 ▶); cell refinement: *SAINT* (Bruker, 2007 ▶); data reduction: *SAINT*; program(s) used to solve structure: *SHELXS97* (Sheldrick, 2008 ▶); program(s) used to refine structure: *SHELXL97* (Sheldrick, 2008 ▶); molecular graphics: *X-SEED* (Barbour, 2001 ▶); software used to prepare material for publication: *publCIF* (Westrip, 2009 ▶).

## Supplementary Material

Crystal structure: contains datablocks global, I. DOI: 10.1107/S1600536809046388/ci2963sup1.cif

Structure factors: contains datablocks I. DOI: 10.1107/S1600536809046388/ci2963Isup2.hkl

Additional supplementary materials:  crystallographic information; 3D view; checkCIF report

## Figures and Tables

**Table 1:** Selected bond lengths (Å)

Mn1—O1	2.106 (2)
Mn1—O4^i^	2.137 (2)
Mn1—O5	2.190 (2)
Mn1—O6	2.545 (2)
Mn1—O7^ii^	2.182 (2)
Mn1—O1*W*	2.157 (2)
Mn2—O2	2.125 (2)
Mn2—O3^iii^	2.173 (2)
Mn2—O5	2.192 (2)
Mn2—O8^ii^	2.104 (2)
Mn2—N1	2.273 (2)
Mn2—N2	2.268 (2)

Symmetry codes: (i) 
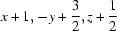
; (ii) 
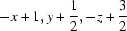
; (iii) 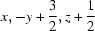.

**Fig. 1.**
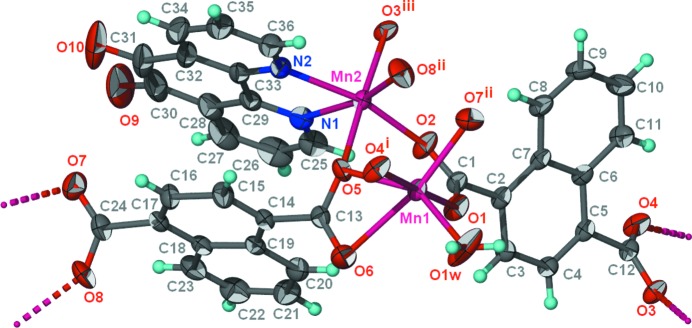
Displacement ellipsoid plot (Barbour, 2001) of a part of the polymeric three-dimensional structure of Mn2(H2O)(C12H6O4)2(C12H6N2O2) at the 50% probability level; H atoms are drawn as spheres of arbitrary radius. Symmetry codes are given in Table 1.

## supplementary crystallographic information

### Experimental

Manganese dichloride tetrahydrate (0.5 mmol, 0.099 g), naphthalene-1,4-dicarboxylic acid (0.5 mmol, 0.108 g), phenanthrene-9,10-dione (0.5 mmol, 0.104 g) and water (12 ml) were heated in a Teflon-lined, stainless-steel Parr bomb at 433 K for 3 days. Yellow crystals were isolated from the cool bomb in 40% yield.

### Refinement

Carbon-bound H-atoms were placed in calculated positions (C-H = 0.93 Å) and were included in the refinement in the riding model approximation, with *U_iso_*(H) set to 1.2*U_eq_*(C). The water H atoms were located in a difference Fourier map, and were refined with distance restraints of O–H = 0.85 (1) Å and H···H 1.39 (1) Å; their U_iso_ values were set to 1.5*U_eq_*(O).

### Crystal data

**Table:**

[Mn_2_(C_12_H_6_O_4_)_2_(C_12_H_6_N_2_O_2_)(H_2_O)]	*F*(000) = 1552
*M**_r_* = 766.42	*D*_x_ = 1.663 Mg m^−^^3^
Monoclinic, *P*2_1_/*c*	Mo *K*α radiation, λ = 0.71073 Å
Hall symbol: -P 2ybc	Cell parameters from 5446 reflections
*a* = 8.4393 (8) Å	θ = 2.1–25.7°
*b* = 19.2477 (18) Å	µ = 0.90 mm^−^^1^
*c* = 19.2504 (19) Å	*T* = 293 K
β = 101.781 (1)°	Block, yellow
*V* = 3061.1 (5) Å^3^	0.27 × 0.26 × 0.20 mm
*Z* = 4	

### Data collection

**Table:**

Bruker APEXII area-detector diffractometer	6015 independent reflections
Radiation source: fine-focus sealed tube	4853 reflections with *I* > 2σ(*I*)
graphite	*R*_int_ = 0.025
φ and ω scans	θ_max_ = 26.1°, θ_min_ = 1.5°
Absorption correction: multi-scan (*SADABS*; Sheldrick, 1996)	*h* = −10→10
*T*_min_ = 0.794, *T*_max_ = 0.841	*k* = −15→23
16986 measured reflections	*l* = −23→22

### Refinement

**Table:**

Refinement on *F*^2^	Primary atom site location: structure-invariant direct methods
Least-squares matrix: full	Secondary atom site location: difference Fourier map
*R*[*F*^2^ > 2σ(*F*^2^)] = 0.036	Hydrogen site location: inferred from neighbouring sites
*wR*(*F*^2^) = 0.096	H atoms treated by a mixture of independent and constrained refinement
*S* = 1.03	*w* = 1/[σ^2^(*F*_o_^2^) + (0.0581*P*)^2^ + 0.1115*P*] where *P* = (*F*_o_^2^ + 2*F*_c_^2^)/3
6015 reflections	(Δ/σ)_max_ = 0.001
466 parameters	Δρ_max_ = 0.40 e Å^−^^3^
3 restraints	Δρ_min_ = −0.24 e Å^−^^3^

### Fractional atomic coordinates and isotropic or equivalent isotropic displacement parameters (Å^2^)

**Table:**

	*x*	*y*	*z*	*U*_iso_*/*U*_eq_	
Mn1	0.67563 (4)	0.723418 (17)	0.635261 (16)	0.02973 (10)	
Mn2	0.32185 (4)	0.688473 (16)	0.694983 (16)	0.02825 (10)	
O1	0.49411 (18)	0.71363 (9)	0.54284 (8)	0.0435 (4)	
O2	0.2654 (2)	0.69971 (10)	0.58282 (8)	0.0491 (4)	
O3	0.09036 (18)	0.76369 (9)	0.19849 (8)	0.0381 (4)	
O4	−0.14651 (18)	0.76639 (9)	0.23115 (8)	0.0448 (4)	
O5	0.55827 (18)	0.64436 (7)	0.68920 (9)	0.0371 (4)	
O6	0.7185 (2)	0.59280 (10)	0.63001 (11)	0.0591 (5)	
O7	0.4054 (2)	0.32857 (10)	0.84788 (10)	0.0574 (5)	
O8	0.5400 (2)	0.27848 (8)	0.77449 (11)	0.0557 (5)	
O9	0.0460 (3)	0.40503 (13)	0.83873 (16)	0.1069 (9)	
O10	0.2619 (3)	0.45832 (15)	0.95212 (15)	0.1063 (10)	
O1W	0.8607 (2)	0.75106 (16)	0.57777 (10)	0.0787 (7)	
H1W1	0.949 (3)	0.754 (2)	0.6082 (16)	0.118*	
H1W2	0.851 (4)	0.7853 (14)	0.5499 (17)	0.118*	
N1	0.1895 (2)	0.58539 (10)	0.69258 (11)	0.0396 (5)	
N2	0.3748 (2)	0.64538 (10)	0.80699 (10)	0.0356 (4)	
C1	0.3440 (3)	0.71125 (12)	0.53511 (11)	0.0334 (5)	
C2	0.2493 (2)	0.72461 (11)	0.46106 (11)	0.0306 (5)	
C3	0.2775 (3)	0.68379 (11)	0.40590 (11)	0.0328 (5)	
H3	0.3502	0.6470	0.4151	0.039*	
C4	0.1973 (2)	0.69744 (11)	0.33599 (11)	0.0303 (5)	
H4	0.2198	0.6703	0.2993	0.036*	
C5	0.0864 (2)	0.74994 (12)	0.32073 (10)	0.0291 (5)	
C6	0.0551 (2)	0.79415 (12)	0.37652 (11)	0.0312 (5)	
C7	0.1407 (2)	0.78174 (11)	0.44707 (11)	0.0310 (5)	
C8	0.1222 (3)	0.82974 (13)	0.50106 (12)	0.0406 (6)	
H8	0.1785	0.8225	0.5473	0.049*	
C9	0.0237 (3)	0.88607 (14)	0.48629 (14)	0.0512 (7)	
H9	0.0149	0.9174	0.5221	0.061*	
C10	−0.0644 (3)	0.89695 (15)	0.41734 (14)	0.0543 (7)	
H10	−0.1336	0.9349	0.4079	0.065*	
C11	−0.0502 (3)	0.85250 (13)	0.36358 (13)	0.0434 (6)	
H11	−0.1099	0.8605	0.3181	0.052*	
C12	0.0036 (3)	0.76148 (11)	0.24480 (11)	0.0303 (5)	
C13	0.6211 (3)	0.58948 (11)	0.66943 (12)	0.0335 (5)	
C14	0.5764 (3)	0.52226 (11)	0.69999 (12)	0.0352 (5)	
C15	0.6256 (3)	0.51188 (12)	0.77124 (13)	0.0437 (6)	
H15	0.6830	0.5467	0.7990	0.052*	
C16	0.5917 (3)	0.44982 (12)	0.80367 (13)	0.0410 (6)	
H16	0.6303	0.4433	0.8520	0.049*	
C17	0.5020 (3)	0.39879 (11)	0.76437 (12)	0.0341 (5)	
C18	0.4411 (3)	0.40882 (11)	0.69041 (12)	0.0342 (5)	
C19	0.4819 (3)	0.47085 (11)	0.65730 (12)	0.0347 (5)	
C20	0.4218 (3)	0.47993 (14)	0.58366 (13)	0.0462 (6)	
H20	0.4474	0.5201	0.5614	0.055*	
C21	0.3261 (3)	0.43017 (15)	0.54474 (14)	0.0551 (7)	
H21	0.2903	0.4361	0.4961	0.066*	
C22	0.2825 (3)	0.37081 (15)	0.57776 (15)	0.0556 (7)	
H22	0.2150	0.3381	0.5510	0.067*	
C23	0.3369 (3)	0.35979 (13)	0.64828 (14)	0.0461 (6)	
H23	0.3058	0.3199	0.6693	0.055*	
C24	0.4799 (3)	0.33048 (12)	0.79864 (13)	0.0393 (5)	
C25	0.0982 (3)	0.55711 (14)	0.63501 (15)	0.0518 (7)	
H25	0.0897	0.5800	0.5919	0.062*	
C26	0.0150 (3)	0.49487 (16)	0.63629 (19)	0.0671 (9)	
H26	−0.0466	0.4762	0.5949	0.081*	
C27	0.0261 (4)	0.46209 (15)	0.6996 (2)	0.0687 (9)	
H27	−0.0283	0.4204	0.7019	0.082*	
C28	0.1182 (3)	0.49066 (13)	0.76087 (17)	0.0523 (7)	
C29	0.1999 (3)	0.55246 (11)	0.75504 (13)	0.0383 (5)	
C30	0.1294 (4)	0.45708 (16)	0.8302 (2)	0.0719 (10)	
C31	0.2429 (3)	0.48801 (17)	0.89331 (19)	0.0677 (9)	
C32	0.3262 (3)	0.55354 (14)	0.88447 (15)	0.0497 (7)	
C33	0.3028 (3)	0.58499 (12)	0.81738 (13)	0.0377 (5)	
C34	0.4268 (4)	0.58562 (17)	0.94055 (15)	0.0600 (8)	
H34	0.4424	0.5663	0.9857	0.072*	
C35	0.5043 (3)	0.64632 (16)	0.92973 (14)	0.0558 (7)	
H35	0.5750	0.6679	0.9668	0.067*	
C36	0.4743 (3)	0.67442 (13)	0.86218 (13)	0.0436 (6)	
H36	0.5261	0.7156	0.8548	0.052*	

### Atomic displacement parameters (Å^2^)

**Table:**

	*U*^11^	*U*^22^	*U*^33^	*U*^12^	*U*^13^	*U*^23^
Mn1	0.02579 (17)	0.0373 (2)	0.02387 (18)	−0.00187 (13)	−0.00003 (13)	0.00353 (13)
Mn2	0.02932 (18)	0.02970 (19)	0.02565 (18)	−0.00156 (13)	0.00545 (13)	0.00262 (13)
O1	0.0295 (8)	0.0699 (12)	0.0270 (9)	0.0000 (8)	−0.0040 (7)	0.0044 (8)
O2	0.0406 (9)	0.0801 (13)	0.0252 (9)	0.0001 (9)	0.0033 (7)	0.0107 (8)
O3	0.0312 (8)	0.0585 (11)	0.0239 (8)	−0.0072 (7)	0.0039 (6)	0.0004 (7)
O4	0.0277 (8)	0.0786 (13)	0.0251 (8)	0.0072 (8)	−0.0019 (6)	0.0002 (8)
O5	0.0374 (8)	0.0267 (8)	0.0477 (10)	0.0047 (6)	0.0099 (7)	0.0071 (7)
O6	0.0663 (12)	0.0533 (12)	0.0682 (13)	0.0105 (9)	0.0383 (11)	0.0109 (9)
O7	0.0712 (13)	0.0504 (11)	0.0552 (12)	−0.0088 (9)	0.0237 (10)	0.0068 (9)
O8	0.0668 (12)	0.0262 (9)	0.0721 (13)	0.0113 (8)	0.0099 (10)	0.0051 (8)
O9	0.114 (2)	0.0741 (17)	0.139 (3)	−0.0251 (15)	0.0409 (19)	0.0406 (16)
O10	0.0751 (16)	0.128 (2)	0.116 (2)	0.0035 (15)	0.0201 (15)	0.0892 (19)
O1W	0.0413 (11)	0.160 (2)	0.0328 (11)	−0.0267 (13)	0.0040 (9)	0.0165 (12)
N1	0.0353 (10)	0.0389 (11)	0.0448 (12)	−0.0035 (8)	0.0086 (9)	−0.0023 (9)
N2	0.0357 (10)	0.0387 (11)	0.0330 (11)	0.0031 (8)	0.0083 (8)	0.0026 (8)
C1	0.0356 (12)	0.0398 (13)	0.0233 (11)	0.0000 (9)	0.0026 (9)	0.0007 (9)
C2	0.0242 (10)	0.0420 (13)	0.0235 (11)	−0.0037 (9)	−0.0001 (8)	0.0032 (9)
C3	0.0305 (11)	0.0364 (13)	0.0293 (12)	0.0020 (9)	0.0007 (9)	0.0014 (9)
C4	0.0304 (11)	0.0338 (12)	0.0252 (11)	−0.0024 (9)	0.0020 (9)	−0.0028 (9)
C5	0.0248 (10)	0.0380 (12)	0.0226 (11)	−0.0055 (9)	0.0001 (8)	0.0018 (9)
C6	0.0232 (10)	0.0442 (13)	0.0258 (11)	0.0001 (9)	0.0037 (8)	0.0008 (9)
C7	0.0240 (10)	0.0448 (14)	0.0236 (11)	−0.0022 (9)	0.0038 (8)	0.0021 (9)
C8	0.0403 (13)	0.0570 (16)	0.0241 (12)	0.0035 (11)	0.0055 (10)	−0.0014 (10)
C9	0.0518 (15)	0.0654 (18)	0.0377 (15)	0.0130 (13)	0.0122 (12)	−0.0118 (12)
C10	0.0486 (15)	0.0658 (19)	0.0469 (16)	0.0253 (13)	0.0062 (12)	−0.0052 (13)
C11	0.0372 (12)	0.0580 (17)	0.0318 (13)	0.0132 (11)	−0.0002 (10)	0.0000 (11)
C12	0.0310 (11)	0.0355 (12)	0.0219 (11)	−0.0039 (9)	−0.0004 (9)	−0.0004 (9)
C13	0.0337 (11)	0.0319 (12)	0.0332 (12)	0.0033 (9)	0.0031 (10)	0.0039 (9)
C14	0.0400 (12)	0.0270 (12)	0.0389 (13)	0.0067 (9)	0.0091 (10)	0.0036 (9)
C15	0.0567 (15)	0.0306 (13)	0.0404 (14)	−0.0062 (11)	0.0019 (12)	−0.0007 (10)
C16	0.0550 (15)	0.0309 (13)	0.0340 (13)	−0.0014 (11)	0.0022 (11)	0.0038 (10)
C17	0.0378 (12)	0.0262 (12)	0.0384 (13)	0.0066 (9)	0.0077 (10)	0.0020 (9)
C18	0.0360 (12)	0.0272 (12)	0.0388 (13)	0.0066 (9)	0.0062 (10)	−0.0024 (10)
C19	0.0380 (12)	0.0298 (12)	0.0363 (13)	0.0097 (9)	0.0078 (10)	−0.0004 (10)
C20	0.0523 (15)	0.0468 (15)	0.0387 (14)	0.0083 (12)	0.0073 (12)	0.0026 (11)
C21	0.0618 (17)	0.0634 (19)	0.0360 (15)	0.0086 (14)	0.0007 (13)	−0.0053 (13)
C22	0.0544 (16)	0.0535 (17)	0.0525 (17)	0.0014 (13)	−0.0044 (13)	−0.0174 (14)
C23	0.0445 (14)	0.0357 (14)	0.0539 (16)	0.0023 (11)	0.0002 (12)	−0.0077 (11)
C24	0.0377 (12)	0.0315 (13)	0.0448 (15)	−0.0028 (10)	−0.0008 (11)	0.0046 (11)
C25	0.0448 (14)	0.0533 (17)	0.0558 (17)	−0.0035 (12)	0.0062 (13)	−0.0083 (13)
C26	0.0498 (16)	0.0559 (19)	0.091 (3)	−0.0129 (14)	0.0027 (16)	−0.0235 (18)
C27	0.0528 (17)	0.0409 (17)	0.112 (3)	−0.0162 (13)	0.0163 (18)	−0.0035 (18)
C28	0.0421 (14)	0.0373 (14)	0.082 (2)	−0.0015 (11)	0.0219 (14)	0.0063 (14)
C29	0.0315 (11)	0.0315 (12)	0.0556 (16)	0.0040 (9)	0.0176 (11)	0.0029 (11)
C30	0.0629 (19)	0.0480 (18)	0.113 (3)	0.0073 (15)	0.038 (2)	0.0318 (18)
C31	0.0480 (16)	0.072 (2)	0.089 (2)	0.0135 (15)	0.0276 (16)	0.0460 (19)
C32	0.0446 (14)	0.0553 (17)	0.0543 (17)	0.0139 (12)	0.0216 (13)	0.0246 (13)
C33	0.0342 (12)	0.0353 (13)	0.0478 (14)	0.0081 (10)	0.0179 (11)	0.0093 (11)
C34	0.0582 (17)	0.080 (2)	0.0435 (16)	0.0232 (16)	0.0148 (14)	0.0234 (15)
C35	0.0564 (17)	0.070 (2)	0.0379 (15)	0.0158 (15)	0.0016 (13)	0.0003 (13)
C36	0.0443 (14)	0.0463 (15)	0.0389 (14)	0.0045 (11)	0.0057 (11)	−0.0002 (11)

### Geometric parameters (Å, °)

**Table:**

Mn1—O1	2.106 (2)	C8—C9	1.361 (4)
Mn1—O4^i^	2.137 (2)	C8—H8	0.93
Mn1—O5	2.190 (2)	C9—C10	1.398 (4)
Mn1—O6	2.545 (2)	C9—H9	0.93
Mn1—O7^ii^	2.182 (2)	C10—C11	1.367 (3)
Mn1—O1W	2.157 (2)	C10—H10	0.93
Mn2—O2	2.125 (2)	C11—H11	0.93
Mn2—O3^iii^	2.173 (2)	C13—C14	1.501 (3)
Mn2—O5	2.192 (2)	C14—C15	1.364 (3)
Mn2—O8^ii^	2.104 (2)	C14—C19	1.422 (3)
Mn2—N1	2.273 (2)	C15—C16	1.404 (3)
Mn2—N2	2.268 (2)	C15—H15	0.93
O1—C1	1.246 (3)	C16—C17	1.370 (3)
O2—C1	1.258 (3)	C16—H16	0.93
O3—C12	1.265 (2)	C17—C18	1.425 (3)
O3—Mn2^iv^	2.1733 (15)	C17—C24	1.500 (3)
O4—C12	1.244 (3)	C18—C23	1.425 (3)
O4—Mn1^v^	2.1371 (15)	C18—C19	1.428 (3)
O5—C13	1.274 (3)	C19—C20	1.416 (3)
O6—C13	1.229 (3)	C20—C21	1.373 (4)
O7—C24	1.240 (3)	C20—H20	0.93
O7—Mn1^vi^	2.1818 (18)	C21—C22	1.393 (4)
O8—C24	1.254 (3)	C21—H21	0.93
O8—Mn2^vi^	2.1039 (17)	C22—C23	1.358 (4)
O9—C30	1.254 (4)	C22—H22	0.93
O10—C31	1.249 (4)	C23—H23	0.93
O1W—H1W1	0.852 (10)	C25—C26	1.392 (4)
O1W—H1W2	0.844 (10)	C25—H25	0.93
N1—C25	1.330 (3)	C26—C27	1.359 (5)
N1—C29	1.346 (3)	C26—H26	0.93
N2—C36	1.335 (3)	C27—C28	1.387 (4)
N2—C33	1.346 (3)	C27—H27	0.93
C1—C2	1.507 (3)	C28—C29	1.391 (3)
C2—C3	1.380 (3)	C28—C30	1.469 (4)
C2—C7	1.421 (3)	C29—C33	1.470 (3)
C3—C4	1.403 (3)	C30—C31	1.507 (5)
C3—H3	0.93	C31—C32	1.471 (4)
C4—C5	1.367 (3)	C32—C34	1.376 (4)
C4—H4	0.93	C32—C33	1.403 (3)
C5—C6	1.436 (3)	C34—C35	1.375 (4)
C5—C12	1.502 (3)	C34—H34	0.93
C6—C7	1.422 (3)	C35—C36	1.383 (4)
C6—C11	1.422 (3)	C35—H35	0.93
C7—C8	1.423 (3)	C36—H36	0.93
			
O1—Mn1—O4^i^	178.03 (6)	O4—C12—O3	124.03 (19)
O1—Mn1—O1W	93.82 (7)	O4—C12—C5	117.92 (18)
O4^i^—Mn1—O1W	88.06 (7)	O3—C12—C5	118.02 (18)
O1—Mn1—O7^ii^	90.73 (7)	O6—C13—O5	120.8 (2)
O4^i^—Mn1—O7^ii^	88.43 (7)	O6—C13—C14	122.7 (2)
O1W—Mn1—O7^ii^	97.43 (10)	O5—C13—C14	116.39 (19)
O1—Mn1—O5	90.97 (6)	C15—C14—C19	119.9 (2)
O4^i^—Mn1—O5	87.72 (6)	C15—C14—C13	118.1 (2)
O1W—Mn1—O5	149.28 (9)	C19—C14—C13	122.0 (2)
O7^ii^—Mn1—O5	112.85 (6)	C14—C15—C16	121.6 (2)
O8^ii^—Mn2—O2	101.33 (7)	C14—C15—H15	119.2
O8^ii^—Mn2—O3^iii^	95.33 (7)	C16—C15—H15	119.2
O2—Mn2—O3^iii^	88.39 (6)	C17—C16—C15	120.3 (2)
O8^ii^—Mn2—O5	82.81 (7)	C17—C16—H16	119.9
O2—Mn2—O5	90.31 (6)	C15—C16—H16	119.9
O3^iii^—Mn2—O5	177.48 (6)	C16—C17—C18	120.0 (2)
O8^ii^—Mn2—N2	92.59 (7)	C16—C17—C24	119.4 (2)
O2—Mn2—N2	164.37 (7)	C18—C17—C24	120.4 (2)
O3^iii^—Mn2—N2	97.43 (6)	C17—C18—C23	122.3 (2)
O5—Mn2—N2	84.37 (6)	C17—C18—C19	119.3 (2)
O8^ii^—Mn2—N1	165.15 (8)	C23—C18—C19	118.5 (2)
O2—Mn2—N1	93.49 (7)	C20—C19—C14	122.5 (2)
O3^iii^—Mn2—N1	85.94 (7)	C20—C19—C18	118.7 (2)
O5—Mn2—N1	96.29 (6)	C14—C19—C18	118.8 (2)
N2—Mn2—N1	72.58 (7)	C21—C20—C19	120.7 (2)
C1—O1—Mn1	130.60 (14)	C21—C20—H20	119.6
C1—O2—Mn2	135.86 (15)	C19—C20—H20	119.6
C12—O3—Mn2^iv^	133.60 (14)	C20—C21—C22	120.3 (3)
C12—O4—Mn1^v^	134.03 (14)	C20—C21—H21	119.9
C13—O5—Mn1	100.28 (13)	C22—C21—H21	119.9
C13—O5—Mn2	140.78 (14)	C23—C22—C21	121.2 (3)
Mn1—O5—Mn2	105.04 (6)	C23—C22—H22	119.4
C24—O7—Mn1^vi^	111.36 (16)	C21—C22—H22	119.4
C24—O8—Mn2^vi^	142.85 (18)	C22—C23—C18	120.6 (3)
Mn1—O1W—H1W1	107 (3)	C22—C23—H23	119.7
Mn1—O1W—H1W2	122 (3)	C18—C23—H23	119.7
H1W1—O1W—H1W2	110.1 (17)	O7—C24—O8	124.5 (2)
C25—N1—C29	118.2 (2)	O7—C24—C17	119.4 (2)
C25—N1—Mn2	124.95 (18)	O8—C24—C17	116.1 (2)
C29—N1—Mn2	116.78 (15)	N1—C25—C26	123.1 (3)
C36—N2—C33	118.2 (2)	N1—C25—H25	118.5
C36—N2—Mn2	124.99 (16)	C26—C25—H25	118.5
C33—N2—Mn2	116.75 (15)	C27—C26—C25	118.2 (3)
O1—C1—O2	126.5 (2)	C27—C26—H26	120.9
O1—C1—C2	115.88 (18)	C25—C26—H26	120.9
O2—C1—C2	117.64 (19)	C26—C27—C28	120.2 (3)
C3—C2—C7	120.04 (19)	C26—C27—H27	119.9
C3—C2—C1	119.28 (19)	C28—C27—H27	119.9
C7—C2—C1	120.53 (19)	C27—C28—C29	118.1 (3)
C2—C3—C4	120.4 (2)	C27—C28—C30	121.3 (3)
C2—C3—H3	119.8	C29—C28—C30	120.6 (3)
C4—C3—H3	119.8	N1—C29—C28	122.1 (2)
C5—C4—C3	121.3 (2)	N1—C29—C33	116.7 (2)
C5—C4—H4	119.3	C28—C29—C33	121.2 (2)
C3—C4—H4	119.3	O9—C30—C28	122.4 (4)
C4—C5—C6	119.96 (19)	O9—C30—C31	119.5 (3)
C4—C5—C12	118.68 (19)	C28—C30—C31	118.1 (3)
C6—C5—C12	121.32 (19)	O10—C31—C32	121.4 (3)
C7—C6—C11	118.5 (2)	O10—C31—C30	119.5 (3)
C7—C6—C5	118.63 (19)	C32—C31—C30	119.1 (3)
C11—C6—C5	122.7 (2)	C34—C32—C33	118.5 (3)
C6—C7—C2	119.54 (19)	C34—C32—C31	121.8 (3)
C6—C7—C8	118.6 (2)	C33—C32—C31	119.7 (3)
C2—C7—C8	121.8 (2)	N2—C33—C32	121.8 (2)
C9—C8—C7	121.2 (2)	N2—C33—C29	117.1 (2)
C9—C8—H8	119.4	C32—C33—C29	121.1 (2)
C7—C8—H8	119.4	C35—C34—C32	119.9 (2)
C8—C9—C10	120.1 (2)	C35—C34—H34	120.0
C8—C9—H9	120.0	C32—C34—H34	120.0
C10—C9—H9	120.0	C34—C35—C36	118.2 (3)
C11—C10—C9	120.9 (2)	C34—C35—H35	120.9
C11—C10—H10	119.6	C36—C35—H35	120.9
C9—C10—H10	119.6	N2—C36—C35	123.3 (3)
C10—C11—C6	120.7 (2)	N2—C36—H36	118.3
C10—C11—H11	119.7	C35—C36—H36	118.3
C6—C11—H11	119.7		
			
O1w—Mn1—O1—C1	−164.4 (2)	Mn1—O5—C13—O6	−0.3 (3)
O7^ii^—Mn1—O1—C1	−66.9 (2)	Mn2—O5—C13—O6	129.4 (2)
O5—Mn1—O1—C1	46.0 (2)	Mn1—O5—C13—C14	176.44 (16)
O8^ii^—Mn2—O2—C1	51.2 (3)	Mn2—O5—C13—C14	−53.8 (3)
O3^iii^—Mn2—O2—C1	146.4 (2)	O6—C13—C14—C15	111.3 (3)
O5—Mn2—O2—C1	−31.5 (2)	O5—C13—C14—C15	−65.5 (3)
N2—Mn2—O2—C1	−101.3 (3)	O6—C13—C14—C19	−70.4 (3)
N1—Mn2—O2—C1	−127.8 (2)	O5—C13—C14—C19	112.9 (2)
O1—Mn1—O5—C13	86.84 (14)	C19—C14—C15—C16	3.0 (4)
O4^i^—Mn1—O5—C13	−94.63 (14)	C13—C14—C15—C16	−178.6 (2)
O1w—Mn1—O5—C13	−12.3 (2)	C14—C15—C16—C17	−2.5 (4)
O7^ii^—Mn1—O5—C13	178.04 (13)	C15—C16—C17—C18	−0.7 (3)
O1—Mn1—O5—Mn2	−62.92 (7)	C15—C16—C17—C24	174.5 (2)
O4^i^—Mn1—O5—Mn2	115.60 (7)	C16—C17—C18—C23	−175.0 (2)
O1w—Mn1—O5—Mn2	−162.08 (11)	C24—C17—C18—C23	9.8 (3)
O7^ii^—Mn1—O5—Mn2	28.27 (10)	C16—C17—C18—C19	3.3 (3)
O8^ii^—Mn2—O5—C13	−172.7 (2)	C24—C17—C18—C19	−171.9 (2)
O2—Mn2—O5—C13	−71.3 (2)	C15—C14—C19—C20	177.4 (2)
N2—Mn2—O5—C13	94.0 (2)	C13—C14—C19—C20	−0.9 (3)
N1—Mn2—O5—C13	22.2 (2)	C15—C14—C19—C18	−0.3 (3)
O8^ii^—Mn2—O5—Mn1	−44.29 (8)	C13—C14—C19—C18	−178.62 (19)
O2—Mn2—O5—Mn1	57.10 (8)	C17—C18—C19—C20	179.5 (2)
N2—Mn2—O5—Mn1	−137.63 (8)	C23—C18—C19—C20	−2.2 (3)
N1—Mn2—O5—Mn1	150.64 (7)	C17—C18—C19—C14	−2.8 (3)
O8^ii^—Mn2—N1—C25	176.3 (3)	C23—C18—C19—C14	175.6 (2)
O2—Mn2—N1—C25	−7.4 (2)	C14—C19—C20—C21	−177.6 (2)
O3^iii^—Mn2—N1—C25	80.76 (19)	C18—C19—C20—C21	0.0 (3)
O5—Mn2—N1—C25	−98.1 (2)	C19—C20—C21—C22	2.0 (4)
N2—Mn2—N1—C25	179.9 (2)	C20—C21—C22—C23	−1.9 (4)
O8^ii^—Mn2—N1—C29	−0.9 (4)	C21—C22—C23—C18	−0.3 (4)
O2—Mn2—N1—C29	175.46 (16)	C17—C18—C23—C22	−179.4 (2)
O3^iii^—Mn2—N1—C29	−96.40 (16)	C19—C18—C23—C22	2.3 (3)
O5—Mn2—N1—C29	84.77 (16)	Mn1^vi^—O7—C24—O8	−18.2 (3)
N2—Mn2—N1—C29	2.70 (15)	Mn1^vi^—O7—C24—C17	161.82 (17)
O8^ii^—Mn2—N2—C36	−4.72 (19)	Mn2^vi^—O8—C24—O7	−45.2 (4)
O2—Mn2—N2—C36	148.4 (2)	Mn2^vi^—O8—C24—C17	134.8 (2)
O3^iii^—Mn2—N2—C36	−100.44 (18)	C16—C17—C24—O7	64.1 (3)
O5—Mn2—N2—C36	77.79 (18)	C18—C17—C24—O7	−120.7 (3)
N1—Mn2—N2—C36	176.2 (2)	C16—C17—C24—O8	−115.9 (3)
O8^ii^—Mn2—N2—C33	177.08 (15)	C18—C17—C24—O8	59.3 (3)
O2—Mn2—N2—C33	−29.8 (3)	C29—N1—C25—C26	−0.8 (4)
O3^iii^—Mn2—N2—C33	81.36 (15)	Mn2—N1—C25—C26	−177.9 (2)
O5—Mn2—N2—C33	−100.41 (15)	N1—C25—C26—C27	0.9 (4)
N1—Mn2—N2—C33	−1.99 (14)	C25—C26—C27—C28	0.1 (5)
Mn1—O1—C1—O2	−16.1 (4)	C26—C27—C28—C29	−1.2 (4)
Mn1—O1—C1—C2	163.06 (14)	C26—C27—C28—C30	178.7 (3)
Mn2—O2—C1—O1	7.0 (4)	C25—N1—C29—C28	−0.4 (3)
Mn2—O2—C1—C2	−172.07 (16)	Mn2—N1—C29—C28	177.00 (17)
O1—C1—C2—C3	56.6 (3)	C25—N1—C29—C33	179.6 (2)
O2—C1—C2—C3	−124.2 (2)	Mn2—N1—C29—C33	−3.1 (2)
O1—C1—C2—C7	−118.8 (2)	C27—C28—C29—N1	1.3 (4)
O2—C1—C2—C7	60.4 (3)	C30—C28—C29—N1	−178.5 (2)
C7—C2—C3—C4	−1.2 (3)	C27—C28—C29—C33	−178.6 (2)
C1—C2—C3—C4	−176.65 (19)	C30—C28—C29—C33	1.6 (4)
C2—C3—C4—C5	−1.8 (3)	C27—C28—C30—O9	−6.6 (5)
C3—C4—C5—C6	2.5 (3)	C29—C28—C30—O9	173.2 (3)
C3—C4—C5—C12	−179.48 (19)	C27—C28—C30—C31	174.9 (3)
C4—C5—C6—C7	−0.3 (3)	C29—C28—C30—C31	−5.2 (4)
C12—C5—C6—C7	−178.25 (19)	O9—C30—C31—O10	5.6 (5)
C4—C5—C6—C11	175.6 (2)	C28—C30—C31—O10	−175.9 (3)
C12—C5—C6—C11	−2.4 (3)	O9—C30—C31—C32	−173.1 (3)
C11—C6—C7—C2	−178.6 (2)	C28—C30—C31—C32	5.4 (4)
C5—C6—C7—C2	−2.6 (3)	O10—C31—C32—C34	−1.0 (4)
C11—C6—C7—C8	−2.4 (3)	C30—C31—C32—C34	177.7 (3)
C5—C6—C7—C8	173.62 (19)	O10—C31—C32—C33	179.5 (3)
C3—C2—C7—C6	3.4 (3)	C30—C31—C32—C33	−1.9 (4)
C1—C2—C7—C6	178.75 (19)	C36—N2—C33—C32	1.9 (3)
C3—C2—C7—C8	−172.7 (2)	Mn2—N2—C33—C32	−179.80 (17)
C1—C2—C7—C8	2.6 (3)	C36—N2—C33—C29	−177.2 (2)
C6—C7—C8—C9	0.7 (3)	Mn2—N2—C33—C29	1.2 (2)
C2—C7—C8—C9	176.8 (2)	C34—C32—C33—N2	−0.5 (3)
C7—C8—C9—C10	1.3 (4)	C31—C32—C33—N2	179.1 (2)
C8—C9—C10—C11	−1.6 (4)	C34—C32—C33—C29	178.5 (2)
C9—C10—C11—C6	−0.1 (4)	C31—C32—C33—C29	−1.9 (3)
C7—C6—C11—C10	2.2 (4)	N1—C29—C33—N2	1.3 (3)
C5—C6—C11—C10	−173.7 (2)	C28—C29—C33—N2	−178.8 (2)
Mn1^v^—O4—C12—O3	2.2 (4)	N1—C29—C33—C32	−177.8 (2)
Mn1^v^—O4—C12—C5	−175.79 (15)	C28—C29—C33—C32	2.2 (3)
Mn2^iv^—O3—C12—O4	139.82 (19)	C33—C32—C34—C35	−1.4 (4)
Mn2^iv^—O3—C12—C5	−42.2 (3)	C31—C32—C34—C35	179.0 (2)
C4—C5—C12—O4	130.1 (2)	C32—C34—C35—C36	1.8 (4)
C6—C5—C12—O4	−51.9 (3)	C33—N2—C36—C35	−1.4 (3)
C4—C5—C12—O3	−48.0 (3)	Mn2—N2—C36—C35	−179.60 (18)
C6—C5—C12—O3	130.0 (2)	C34—C35—C36—N2	−0.4 (4)

Symmetry codes: (i) *x*+1, −*y*+3/2, *z*+1/2; (ii) −*x*+1, *y*+1/2, −*z*+3/2; (iii) *x*, −*y*+3/2, *z*+1/2; (iv) *x*, −*y*+3/2, *z*−1/2; (v) *x*−1, −*y*+3/2, *z*−1/2; (vi) −*x*+1, *y*−1/2, −*z*+3/2.

### Hydrogen-bond geometry (Å, °)

**Table:**

*D*—H···*A*	*D*—H	H···*A*	*D*···*A*	*D*—H···*A*
O1W—H1W1···O3^i^	0.85 (1)	1.93 (2)	2.720 (2)	155 (3)

Symmetry codes: (i) *x*+1, −*y*+3/2, *z*+1/2.
